# Group A Streptococcus molecular point of care testing in a paediatric emergency department

**DOI:** 10.1093/jac/dkag290

**Published:** 2026-08-31

**Authors:** Ewurabena A Mills, Rebekah Bingham, Ruud G Nijman, Shiranee Sriskandan

**Affiliations:** Department of Infectious Disease, Imperial College London, London, UK; Department of Infectious Disease, Imperial College London, London, UK; Department of Infectious Disease, Imperial College London, London, UK; Department of Paediatric Emergency Medicine, Division of Medicine, St. Mary’s Hospital, Imperial College NHS Healthcare Trust, London, UK; Centre for Paediatrics and Child Health, Imperial College London, London, UK; Department of Infectious Disease, Imperial College London, London, UK; NIHR Health Protection Research Unit in Healthcare Associated Infections and AMR, Department of Infectious Disease, Imperial College London, London, UK; Centre for Bacterial Resistance Biology, Imperial College London, London, UK

## Abstract

**Background:**

An upsurge in *Streptococcus pyogenes* infections 2022–2023 highlighted potential benefits of point-of-care tests (POCT) to support clinical pathways, prevent outbreaks and optimize antibiotic use.

**Objectives:**

We conducted a pilot research study in a west London paediatric emergency department (ED) to determine whether a molecular POCT had potential to alter management in children who were also having a conventional throat swab taken for culture.

**Methods:**

Children <16 years presenting to ED who had a throat swab requested by a clinician were invited to have a second swab taken for research purposes only. Clinical management was unaffected by the research swab result, which was processed using a molecular POCT that was not approved for use in the host NHS Trust.

**Results:**

Prevalence of streptococcal infection was low during the study (May 2023–June 2025); swab positivity in symptomatic children was 12.8% (6/47). Overall, 38/49 (77.6%) participants who had throat swabs received antibiotics. Of those children recommended to receive antibiotics, 29/38 (76.3%) had a negative POCT. Mean time to reporting of positive throat swab culture results was 3.67 days (range 3–5 days) leading to occasional delay in treatment, although POCT identified positive results within minutes.

**Conclusion:**

Antibiotic use was frequent and could be avoided by use of a ‘rule out’ POCT in many children in the ED, provided suspicion of *S. pyogenes* is the main driver for prescribing. POCT were easy to process and produced immediate results compared with culture. More research is needed to establish whether POCT confers overall net benefit.

## Introduction

In England and Wales, national guidance advocates against diagnostic testing for sore throat, instead recommending use of syndromic scoring systems, such as FeverPain or Centor score, to identify patients >5 years who might benefit from antibiotics.^1^ NICE guidance in 2008 advocated a pragmatic no-treatment or deferred treatment approach to sore throat on the basis of syndromic assessment only.^2^ Point of care testing (POCT) for *Streptococcus pyogenes* (group A *Streptococcus,* GAS) is undertaken in many other developed countries in Europe and North America to aid diagnosis.^3,4^ However, predicted minimal effects on reducing antibiotic use have precluded adoption in England and Wales in those aged >5 years,^5^ with no specific evaluation in younger children, where scoring systems are of uncertain value.^6^

A post-pandemic upsurge of *S. pyogenes* in 2022–2023 renewed interest in POCT. In England, there were 58 972 scarlet fever notifications, 3729 invasive GAS infections and 491 deaths, including 55 in children,^7^ alongside increased beta-lactam prescribing^8^ and supply chain pressures. Interim guidance^9^ lowered thresholds for antibiotic use and allowed alternatives under a serious shortage protocol. Public concern as well as disease surge drove major service pressures, with ED attendances rising sharply for sore throat (+953%) and scarlet fever (+2640%) compared with winter means.^10^

To investigate whether the rapid molecular detection of *S. pyogenes* might have potential to optimize ED management of children presenting with sore throat, we undertook a research and feasibility study in west London. Children with suspected infection who had a throat swab taken for culture were invited to have a second swab for research purposes.

## Methods

### Recruitment

The study ran from 25 May 2023 to 25 June 2025. Children aged <16 years presenting to the paediatric ED at Imperial College Healthcare NHS Trust where the attending clinician requested a throat swab for culture were invited to take part in the study. Parents or guardians were approached by the research team to ask if a second (research) swab could be taken. The research was approved by a National Research Ethics Committee (Camden and Kings Cross 14/LO/2217 IRAS 166513).

The original aim was to recruit 200 children during the upsurge period; however, by May 2023 presentations to ED had reduced. The study was therefore terminated after 50 children were recruited for pragmatic reasons.

### Microbiology and other data collected

Routine throat swabs underwent standard culture and reporting in the hospital laboratory. Research swabs from consenting participants were tested separately by a researcher using a molecular POCT (Abbott ID Now). As this test lacked local NHS Trust validation, results were not shared with clinicians and were for research only.

Data collected by researchers included demographics, presentation time, ED stay duration, FeverPAIN and Centor scores, prescriptions and outcomes (e.g. admission or re-presentation). Culture results, including time to result and subsequent prescribing actions, were also recorded.

### Results and discussion

Data from 49 children who had both clinical and POCT swabs taken were included. Clinical swab culture results on two children were missing. The median age was 4.0 years (range 9 months to 15 years) (Table S1, available as Supplementary data at *JAC* Online).

Several observations emerged. Antibiotic prescribing was common (38/49, 77.5%) among children who had a throat swab, probably reflecting concern for bacterial infection (Table 1). Clinicians suspected *S. pyogenes* across a range of presentations in the ED; indications for throat swabbing included sore throat (with or without fever, or with lymphadenitis) (*n* = 24), rash with or without fever or cough (*n* = 11), fever only (*n* = 9) or other (*n* = 5, including lymphadenitis and cough). Cough is considered a negative predictor of *S. pyogenes* in current sore throat scoring algorithms, but may need re-evaluation given the risks of pneumonia/empyema in younger children^7^). In the group with sore throat, ED use of scoring systems appeared limited (Tables S2 and S3).

**Table 1.** Antibiotic decision for whole cohort in the ED

	Received antibiotic	Did not receive antibiotic^a^
Number (% of total)	38 (77.6)	11 (22.4)
Already on antibiotic	11	2
Throat culture positive	5	1
Days to culture result, median (range)	3 (1–7)	3 (1–5)
DNA POCT positive	8	2
DNA POCT fail	1	0
DNA POCT negative	29	9

^a^Includes one patient receiving a prescription for delayed antibiotic.

The overall prevalence of throat swab positivity was 12.8% (6/47) in symptomatic children, consistent with reduced prevalence of streptococcal infection during the study period (May 2023–June 2025). Of the six children who had a positive *S. pyogenes* throat culture, five of them would have been identified with the DNA POCT immediately, compared with a 3–5 day delay waiting for a culture result (Table 2). In one case, the POCT delivered an indeterminate result due to the need for a software upgrade. Of six children with positive throat culture results, all bar one received empiric antibiotics; that child had to be recalled 3 days later when the culture result was received, underlining the value of a rapid result.

**Table 2.** Characteristics of whole cohort by throat culture results^a^

	*S. pyogenes* culture positive	*S. pyogenes* culture negative
Total	6	41
DNA POCT positive	5	5
DNA POCT negative	0	36
DNA POCT fail	1	0
Days to culture result, median (range)	3 (3–5)	3 (1–7)
Given antibiotics	5	31
Not given antibiotics	1	10
Given analgesics	1	21
Unknown	1	6
Not given analgesics	4	14

^a^Excludes two patients with missing results from throat swab culture.

Of the 41 children who had a negative throat culture result, 31/41 (75.6%) received antibiotics initially. However, of those who received antibiotics, 27/31 (87.1%) had a negative DNA POCT test, and might have avoided antibiotic treatment altogether had a POCT been used and the result acted on (Table 2). We cannot exclude the possibility that another type of bacterial infection was suspected in children presenting to the ED, providing an alternate rationale for antibiotic prescribing. Future studies should collect specific indications for antibiotics to better quantify this. Focusing only on the 24 children with sore throat as a presentation (Tables S2 and S3), 19/24 received antibiotics initially, however, of them, 14/19 (74%) had a negative POCT result and would have avoided antibiotics. The time taken for a culture result to be available was highly variable, ranging up to 7 days depending on when the swab was taken, meaning that some children remained on antibiotics for up to 1 week without need.

The POCT was positive in five cases where the routine swab for culture was negative. If culture is considered to be the gold standard, then these might be considered ‘false positives’ and trigger unnecessary prescribing. Discordance could be explained by sampling error; swabs for culture and POCT were not always taken by the same person nor at exactly the same time. Furthermore, a molecular POCT may have greater sensitivity to detect low level *S. pyogenes* carriage than culture alone. Two children with discordant results were taking antibiotics already. A POCT that detects pathogen DNA will remain positive despite a negative culture result, a feature that could be useful to aid decision making about continuation of antibiotics. The remaining discordant results all occurred in June 2023 at a time when the upsurge in *S. pyogenes* was tailing off, potentially reflecting a higher prevalence of infection. After June 2023, there were no further culture-negative, POCT-positive results. The hazard of children with a positive POCT being prescribed unnecessary antibiotics is important to recognize; in this low prevalence setting, the potential benefit of POCT on prescribing appeared to outweigh this hazard. This should be re-evaluated in a high prevalence setting.

It is estimated that in primary care, 59% of consultations for sore throat in England result in prescription of an antibiotic.^11^ Although very different from our study, ‘Sore throat test and treat’ and ‘Pharmacy First’ studies of pharmacy-led POCT in the community have been conducted in Wales and Northern Ireland, respectively.^12,13^ Both studies evaluated use of POCT in patients aged >5 (>6 in Wales) years with a FeverPAIN score of two or more to guide prescribing. Overall, 21%–25% of consultations resulted in an antibiotic prescription, which is much lower than primary care in England, where POCT are not used.^11^ These studies used antigen detection tests, which are cheaper than molecular tests and many do not require a dedicated instrument. A Cochrane review also found that antibiotic prescribing could be reduced by 25% if POCT were used.^14^ A previous implementation study in London used scoring system-directed molecular testing and found that antibiotic use for tonsillitis was less than the previous year.^15^

Trials outside the UK support the use of POCT in paediatric ED.^16^ Our study was conducted to determine feasibility of a larger trial of POCT in a UK ED setting. Performing the molecular test was easy to do and results were obtained within minutes. Of 119 children screened for the study, only 49 were recruited mainly due to reluctance to have a second research-only swab. However, parents were keen to have a diagnostic test. We posit that there would be increased willingness to participate if results were to be provided at the time of swabbing to clinicians and patients.

The pilot study intentionally used a molecular POCT as it was felt that clinicians would wish to use the most sensitive test to effectively rule out *S. pyogenes* infection, although current cost may preclude broader use. Whether a negative antigen test would provide similar assurance is unclear. The results point to a potential net benefit of POCT in reducing unnecessary prescribing at least in this paediatric ED setting, during the period studied. The net impact in other settings remains un-evaluated, including potential for over-diagnosis when using molecular tests. The results also highlight the rapidity of *S. pyogenes* detection compared with conventional culture, a feature that could be of value in optimizing time to treatment, controlling outbreaks, and supporting ED pathways. Economic analyses of different tests are needed to balance testing costs and risks of false positives against value of optimized prescribing, including both reduction of transmission, as well as avoidance of unnecessary antibiotic use.

## Supplementary Material

dkag290_Supplementary_Data

## Data Availability

All data from this study is provided in the associated Tables and supplementary Tables.

## Supplementary data

Tables S1 to S3 are available as Supplementary data at *JAC* Online.
