# Supplementary material for: Group A Streptococcus molecular point of care testing in a paediatric emergency department
Source: J Antimicrob Chemother. 2026 Aug 31;81(9):dkag290. doi: 10.1093/jac/dkag290 (PMC13526505; doi:10.1093/jac/dkag290)
Supplement: Supplementary file 1 [file dkag290_supplementary_data.docx]

**Supplementary File**

**Supplementary methods**

*Study eligibility* Eligibility for the study was any acute presentation to the ED where the attending clinician requested a throat swab for culture based on clinical concern; clinical decision making regarding swabbing and treatment was not influenced by this study. The research study enrolled patients only once the decision to take a routine swab was taken; ED clinicians were not aware of the result of the second (research) swab.

*Paediatric ED routine practice*. At the time of the study, throat swabs were routinely being taken on approximately 20% of children with a final (coded) diagnosis of tonsillitis/pharyngitis. Throat swabs were also taken on children who did not specifically complain of tonsillitis/pharyngitis based on clinical concern and findings.

*Microbiology routine practice.* Swabs transported in Amies medium from the paediatric ED are transported to a specimen hub and then to a diagnostic laboratory at intervals during working hours. Swabs are set up for culture on Columbia blood agar in working hours. Plates are read 24h after incubation; any beta haemolytic colonies are tested by MALDI-ToF (Bruker Biotyper); any *S. pyogenes* are reported on the hospital computer system regardless of growth density after susceptibility testing.

**Supplementary Tables**

**Table S1.** Age distribution across study

| Age Range | Male | Female | Total |
| --- | --- | --- | --- |
| <1 | 2 | 0 | 2 |
| 1-4 | 8 | 15 | 23 |
| 5-9 | 7 | 5 | 12 |
| 10-16 | 6 | 6 | 12 |
|  |  | Total | 49 |

**Table S2.** Antibiotic decision for children presenting with sore throat

|  | **Received antibiotic** | **Did not receive antibiotic** |
| --- | --- | --- |
| Number (% of total) | 19 (79.2) | 5 (20.8) |
| Already on antibiotic | 7 | 1 |
| FeverPAIN* Score median (range) | 3 (1-4) | 3 (1-3) |
| Centor* Score median (range) | 2 (0-3) | 2 (1-3) |
| Throat culture positive | 1 | 1 |
| Days to culture result, median (range) | 3 (1-7) | 4 (2-5) |
| DNA POCT positive | 4 | 1 |
| DNA POCT fail | 1 | 0 |
| DNA POCT negative | 14 | 4 |

*FeverPAIN and Centor scores collate clinical signs and symptoms. NICE guidance advocates antibiotics to be considered for Centor ≥3 (out of 4) or FP ≥4 (out of 5) in people >5 years of age

**Table S3.** Characteristics of children presenting with sore throat by throat culture results

|  | ***S. pyogenes* culture positive** | ***S. pyogenes* culture negative** |
| --- | --- | --- |
| Total | 2 | 22 |
| DNA POCT positive | 1 | 4 |
| DNA POCT negative | 0 | 18 |
| DNA POCT failed | 1 | 0 |
| Days to culture result, median (range) | 4 (3-5) | 3 (1-7) |
| FeverPAIN*score, median (range) | 3 (3-3) | 3 (1-4) |
| Centor* score, median (range) | 2 (2-2) | 2 (0-3) |
| Given antibiotics | 1 | 18 |
| Not given antibiotics | 1 | 4 |
| Given analgesics | 1 | 14 |
| Unknown | 0 | 2 |
| Not given analgesics | 1 | 6 |

*FeverPAIN and Centor scores collate clinical signs and symptoms. NICE guidance advocates antibiotics to be considered for Centor ≥3 (out of 4) or FP ≥4 (out of 5) in people >5 years of age
